# A Case of Congenital Lockjaw

**Published:** 1890-11

**Authors:** 


### A Case of Congenital Lockjaw.

No definite history could be obtained concerning this boy except that he was five years of age, and that nothing unusual had been noticed about the jaw until a short time ago. The boy was quite intelligent and no other joints were affected. The jaw appeared to be subluxated backward and the deformity was presumably congenital. The recession of the jaw and the apparent atrophy on both sides added to the interest of the case. Dr. Sayre said that before adopting any operative measures he would attempt to relieve the case by stretching, and for this purpose would employ a wedge-shaped instrument devised by Dr. L. W. Hubbard, and presented last year before the Society of the Alumni of Bellevue Hospital. It consisted of two plates of steel fastened together by a separable hinge, and capable of being separated at the other end by turning a screw. Having partly separated the jaws of the instrument, a cork could be inserted between the plates near the hinge, and the action of the screw reversed when the instrument would exert considerable pressure. —*Buffalo Med. and Sur. Journal.*

